# Changes in Physico-Chemical and Storage Properties of Dry-Aged Beef Loin Using Electric Field Refrigeration System

**DOI:** 10.3390/foods11111539

**Published:** 2022-05-24

**Authors:** Kyu-Min Kang, Sol-Hee Lee, Hack-Youn Kim

**Affiliations:** Department of Animal Resources Science, Kongju National University, Yesan 32439, Chungnam, Korea; rbals15@naver.com (K.-M.K.); chzh73@naver.com (S.-H.L.)

**Keywords:** beef loin, dry aging, electric field refrigerate system, physico-chemical properties, microbial properties

## Abstract

The aim of this study is to establish the dry aging period of beef loin in an electric field refrigeration system. Beef loins (Korea quality grade 2) were dry aged at 0, −1, and −2 °C temperature in an electric field refrigeration system (air velocity, 5 ± 2 m/s) and aging stopped as the value of TPC reached 7 log CFU/g. Samples were examined by aging yield, trimming yield, pH, color, water holding capacity (WHC), cooking yield, shear force, total plate count (TPC), 2-thiobarbituric acid reactive substances (TBARS), and volatile basic nitrogen (VBN). The results for aging yield, trimming yield, redness, yellowness, and chroma decreased with increasing the dry aging period. Contrariwise, those for pH, lightness, hue angle, WHC, and cooking yield increased with the dry aging period. In shear force, the lowest value occurred at four weeks at all temperatures. The results for TPC, TBARS, and VBN increased with aging period, and VBN at 6 weeks at 0 °C and 9 weeks at −1 °C exceed the standard value (20 mg/100 g), while dry aging temperature had an effect on physico-chemical and storage properties by lower temperatures showed slower progress. Therefore, dry aging on an electric field refrigerate system can be used until 4 weeks at 0 °C, 8 weeks at −1 °C, and 10 weeks at −2 °C. However, considering physico-chemical properties, 4 weeks at every temperature is suitable for manufacturing soft dry-aged beef loin.

## 1. Introduction

In South Korea, the standard beef quality grades are divided into grades 1^++^, 1^+^, 1, 2, and 3 based on marbling (grades 1 to 9) [1]. Grades 1^++^ and 1^+^ show a prevalence rate of 30.7%, but their consumer preference is 73.7%, which is quite high [2]. Most of the low-grade beef with a low consumer preference rating is Holstein, which is a major livestock breed consumed in South Korea along with Hanwoo (a breed of cattle native to Korea) [3,4]. Holstein beef has a relatively high protein content despite its low marbling content, it has potential value as a food ingredient that meets the needs of modern people who prefer high-protein, low-fat foods [5]. Therefore, the texture of Holstein meat needs to be improved as the consumers prefer, and to solve this problem, various techniques such as aging, brining, and physical tenderization are applied [6,7].

Aging is a typical method of improving the sensory quality of meat, and the texture is effectively enhanced through complex changes in muscle metabolism after slaughtering [8]. Dry aging is the process of aging the meat by exposing the meat surface to the air, which leads to the hydrolysis of proteins and fat by bacteria to quickly improve the tenderness of the meat [9]. Generally, dry aging takes place between 0 °C and 4 °C, but long-term aging is difficult because microorganisms on the meat surface produce peroxides and aldehydes [10]. At low temperatures (−1 °C to −3 °C), on the other hand, the water content in the meat freezes, which increases the volume of the water molecules, destroying muscle. When thawed, it leads to a large amount of drip loss, along with decreases in qualities such as water holding capacity (WHC), texture, and sensory properties [11].

In an electric field refrigeration system, the water molecules contained in the food vibrate in the direction of the electric field they are exposed to, and as a result, a hygienically safe state can be maintained without freezing at a temperature below the freezing point [12]. Refrigeration systems have been reported to be a promising technology that can maintain the freshness of foods by inhibiting microbial growth at low temperatures [13]. Furthermore, electric field refrigeration systems can have a positive effect on meat by increasing flavor, yield, and storage properties through physical and biochemical changes in a hygienically safe state [14]. Accordingly, few studies have been conducted in which electric fields are applied to meat [15].

In South Korea, however, the electric field dry-aging standard is unclear, and there are very few cases of the application of electric field refrigeration systems [16]. Therefore, we aim to use this study to provide basic data to establish an electric field dry-aging system using an electric field refrigeration system at temperatures below freezing point.

## 2. Materials and Methods

### 2.1. Preparation of Dry-Aged Beef Loin

Beef loin (Holstein; M. *longissimus dorsi*; Korea quality grade 2; Ihome meat, Seoul, Korea) was refrigerated for 24 h after slaughter, and loins were cold delivered within 12 h and used after removing excess fat and connective tissues. For the experiment, beef loins were collected from a total of 27 carcasses. The loins of the carcasses were grouped into three groups and then severed in 290–310 g portions and randomly placed in an electric field refrigerator system [10] (air velocity, 5 ± 2 m/s; electric field strength, 5 kV; ARD-090RM-F, Mars, Fukushima, Japan) at three temperatures, 0 °C, −1 °C, −2 °C, and aging stopped as the value of TPC reached 7 log CFU/g. After aging, samples were trimmed for experiments, and the crust (surface of dry-aged beef loin) was examined for storage properties and the inner edible parts for physico-chemical properties.

### 2.2. Aging Yield

The aging yield of the samples was determined from the weights both before and after dry aging with the following formula [17].

$$\mathrm{Aging}\;\mathrm{yield}\;\left(\%\right)=\frac{\;\mathrm{Sample}\;\mathrm{weight}\;\mathrm{after}\;\mathrm{aging}\;(g)}{\mathrm{Sample}\;\mathrm{weight}\;\mathrm{before}\;\mathrm{aging}\;(g)}\text{×}100$$

### 2.3. Trimming Yield

The trimming yield of the samples was determined from the weights both before and after trimming the surface of dry-aged beef loins (crust) with the following formula [17].

$$\mathrm{Trimming}\;\mathrm{yield}\;\left(\%\right)=\frac{\;\mathrm{Sample}\;\mathrm{weight}\;\mathrm{after}\;\mathrm{trimming}\;(g)}{\mathrm{Sample}\;\mathrm{weight}\;\mathrm{after}\;\mathrm{aging}\;(g)}\text{×}100$$

### 2.4. pH

The samples were homogenized with distilled water (1:4, *v*/*v*) using an Ultra Turrax homogenizer (HMZ-20DN, Poonglim Tech, Seongnam, Korea) for 1 min at 6991× *g*. After homogenizing, the pH of the mixture was determined using a pH meter (Model S220, Mettler-Toledo, Columbus, OH, USA). Prior to the analysis, the pH meter was calibrated in room temperature using pH buffer solutions of pH 4.01 ± 0.01, pH 7.00 ± 0.01, and pH 10.00 ± 0.01 (Suntex Instruments co. ltd., New Taipei City, Taiwan).

### 2.5. Color

After dry aging finished, the samples’ cores were cut into 5 × 5 × 5 cm^3^ blocks right away and surfaces were randomly evaluated using a colorimeter, adjusted to operate with an aperture of 8 mm, 2° standard observer, illuminant D65, and pulsed xenon lamp as a default light source. Before measuring, the device was calibrated with a white plate, CIE L*: +97.83, CIE a*: −0.43, and CIE b*: +1.98 (CR-10, Minolta, Tokyo, Japan); the lightness (CIE L*), redness (CIE a*), and yellowness (CIE b*) were recorded. The Hue angle and Chroma value were calculated with the following formula.

$${\mathrm{Hue}\;\mathrm{angle}=\tan}^{-1}b^{*}{/a}^{*},\;\mathrm{Chroma}={\left(a^{*2}{+b}^{*2}\right)}^{\frac{1}{2}}$$

### 2.6. Water Holding Capacity (WHC)

The WHC of samples was determined by the filter paper press method [18]. An amount of 0.3 g of dry-aged beef loin inner part was placed at the center of the filter paper (Whatman No. 1, GE Healthcare, Chicago, IL, USA) and compressed for 3 min using a plexiglass plate device. The WHC was calculated with the following formula.

$$\mathrm{WHC}\;\left(\%\right)=\frac{\;\mathrm{Meat}\;\mathrm{area}\;({\mathrm{mm}}^{2})}{\mathrm{Exudation}\;\mathrm{area}\;({\mathrm{mm}}^{2})}\text{×}100$$

### 2.7. Cooking Yield

The cooking yield of the samples was determined from the weights before and after cooking (core temperature 72 ± 1 °C for 120 min by convection) and then after cooling at 10 °C for 20 min with the following formula [19].

$$\mathrm{Cooking}\;\mathrm{yield}\;\left(\%\right)=\frac{\;\mathrm{Sample}\;\mathrm{weight}\;\mathrm{after}\;\mathrm{cooking}\;(g)}{\mathrm{Sample}\;\mathrm{weight}\;\mathrm{before}\;\mathrm{cooking}\;(g)}\text{×}100$$

### 2.8. Shear Force

Samples were cut into 1 × 1 × 1 cm^3^ blocks and each block was analyzed using a texture analyzer (TA 1, Lloyd, Largo, FL, USA); the machine analyzing conditions were as follows: V-blade with a test speed of 21.0 mm/s, a head speed of 21.0 mm/s, a distance of 22.0 mm, and a force of 5.6 N. Measured values are expressed in Newtons (N).

### 2.9. Total Plate Count (TPC)

A sample of 10–20 g of crust sample was placed into sample bags (193OF, 3M, Saint Paul, MN, USA) with 0.1% buffer peptone water twice the weight of samples. After measuring, samples were stomached in a stomacher (WH4000-2751-9, 3M, Saint Paul, MN, USA) for 2 min and prepared by repeating as many dilutions as necessary. Diluted samples were plated in typic soy agar (BD Difco, Franklin Lakes, NJ, USA) and incubated at 37 °C in an incubator (WSC-2610, ATTO, Tokyo, Japan) for 24 h. Counts were recorded as colony forming units per gram (CFU/g).

### 2.10. Thiobarbituric Acid Reactive Substances (TBARS)

Lipid oxidation degree of samples were determined using the TBARS method of Witte et al. [20]. Five grams of crust sample, 12.5 mL of 10% PCA solution, and 200 µL of 0.3% BHT were homogenized using homogenizer (AM-5, Nihonseiki, Tokyo, Japan) for 1 min and filtered with filter paper (Whatman No. 1, GE Healthcare), then 5 mL of filtrate was mixed with 0.02 M TBA solution and reacted in a 100 °C water bath (JSWB-30T, JSR, Gongju, Korea) for 10 min. Measurements were then made using a multi-mode microplate reader (Spectra Max iD3, Molecular devices, San Jose, CA, USA) at an absorbance of 532 nm. The amount of malondialdehyde (MDA) was calculated using a standard curve prepared from 1,1,3,3-trethoxypropane, and the TBARs value was reported as mg MDA per kg of sample.

### 2.11. Volatile Basic Nitrogen (VBN)

VBN was determined using the method of Choi et al. [21]. Ten grams of crust sample was weighted with 30 mL of distilled water and homogenized at 10,923× *g* for 1 min. After homogenizing, it was massed with distilled water to 100 mL and filtered through filter paper (Whatman No. 1, GE Healthcare). The outer chamber of the Conway unit was filled with 1 mL of filtrate and the inner chamber was filled with 1 mL of 0.01 N H_3_BO_3_, then 100 µL of Conway reagent was put in the inner chamber and 1 mL of 50% K_2_CO_3_ was put in the outer chamber and sealed. The unit was incubated at 37 °C for 2 h, after which the collected liquid of the inner chamber was titrated with 0.02 N H_2_SO_4_ and calculated using the following formula:

VBN(mg/100g)=(A-B) × (f × 0.02N × 14.007 × 100 × c)/S

where A is the volume of sulfuric acid consumed for the sample titration (mL), B is the volume of sulfuric acid consumed for the blank titration (mL), f is factor of reagent, N is normality, c is dilution ratio, and S is sample weight

### 2.12. Statistical Analysis

All experimental results were assessed after a minimum of three repeated trials. Statistical analyses were performed using SAS (version 9.3 for window, SAS Institute Inc., Cary, NC, USA) at a confidence level of *p* < 0.05; results are indicated herein as a mean and standard error of the means (SEM). Data were arranged by two different factors (temperature and aging period) and significant differences were verified using a one-way analysis of variance (ANOVA) and Duncan’s multiple range tests.

The general linear model (GLM) of the ANOVA was used to determine, separately, the significant differences in the aging yield, trimming yield, pH, color, WHC, cooking yield, shear force, TPC, TBARS, and VBN measurements of beef loin samples among aging periods and temperatures.

## 3. Results and Discussion

### 3.1. Aging Yield and Trimming Yield

Table 1 shows the aging yield and trimming yield of beef loin, which was dry aged using the electric field refrigeration system. The aging yield showed a decreasing trend as the aging period increased at all temperatures. In the second, fourth, and sixth weeks, a significantly higher aging yield was shown at 0 °C than at −1 °C and −2 °C (*p* < 0.05). This is because dry aging leads to extensive water evaporation due to the surface being exposed to the air, and, consequently, a thick crust is produced on the surface, which affects the aging yield [17]. As the internal water content of the meat evaporates, the aging yield decreases, and when there are no elements that prevent contraction, such as bones and intermuscular fat, the aging yield decreases more rapidly [22]. The trimming yield showed a decreasing trend as the aging period increased at all temperatures. In the second, seventh, eighth, and ninth weeks of aging, the trimming yield was significantly higher at −2 °C than at −1 °C (*p* < 0.05). This is because in the case of dry aging, the surface is exposed to the air, and as the temperature drops, the contraction rate of the muscles slows down, which decreases the drainage rate of the internal water content to the outside [23]. If the water content drainage rate decreases, the reduction of the water content also slows down, and the hardening of the meat surface proceeds more slowly [24]. Therefore, if the electric field refrigeration system is used to proceed with dry aging, hardening will occur more slowly as the temperature decreases, producing a larger edible part.

### 3.2. pH and Color

Table 2 shows the pH values of beef loin dry aged using the electric field refrigeration system. pH showed an increasing trend as the aging period increased at all temperatures, and it increased more slowly as the aging temperature dropped. The rise in pH with an increase in the dry-aging period is due to the alkaline substances produced by the proteolytic activity of micro-organisms [25]. The proteolytic activity of micro-organisms is less active at low temperatures, leading to slower production of alkaline substances, which affects pH [26]. Because a rise in pH inhibits the oxidation of myoglobin, it can minimize the discoloration of aged meat [27], and it is determined that if dry aging is performed at low temperatures, as in this study, the discoloration rate will decrease, having a positive effect on the quality.

Table 3 shows the color values of the beef loin dry aged using the electric field refrigeration system. The lightness value (CIE L*) showed an increasing trend as the aging period increased at all temperatures. The lightness has a positive correlation with pH, and if pH increases, the length of the sarcomeres increases, which lightens the dark segments, affecting lightness [28]. Furthermore, as the space between muscle fibers increases due to the increase in pH, the light absorption rate increases along with a decrease in the reflection rate, resulting in a higher lightness [29]. On the other hand, the redness (CIE a*) and yellowness (CIE b*) tended to decrease as the aging period increased at all temperatures. It seems that as oxygen and the myoglobin of aged meat bond, they form oxidized myoglobin, affecting the redness and yellowness [30]. When the heme pigment of myoglobin bonds with oxygen, the redness and yellowness of the aged meat decrease, and in the case of dry-aged meat, the oxidation reaction is greater because the area exposed to the air is large [31]. At all temperatures, as the aging period increased, the hue angle showed an increasing trend, and the chroma showed a decreasing trend. Hue angle and chroma are affected by the redness: if the redness decreases, the chroma decreases and the hue angle increases [32]. The metmyoglobin concentration is used as an indicator of the degree of discoloration of meat based on the correlations of redness, hue angle, and chroma [33]. In this study, we have confirmed that at all temperatures, as the aging period increases, the lightness and hue angle of beef increase, and the redness, yellowness, and chroma decrease, resulting in discoloration. Furthermore, it is determined that dry-aged meat having a similar chromaticity as fresh meat can be produced at low temperatures since discoloration will occur slowly.

### 3.3. WHC, Cooking Yield, and Shear Force

Table 4 shows the WHC values of the beef loin dry aged using the electric field refrigeration system. WHC showed an increasing trend as the aging period increased at all temperatures. In meat, WHC has a positive correlation with pH [34], and this study also showed that the pH and WHC of the meat dry aged using the electric field refrigeration system have a positive correlation. As the pH increases, the number of anion increased and it spread the gap between muscle fibers, which increases the space that can store water, resulting in a higher WHC [35]. Furthermore, as the aging period of dry-aged meat increases, the free water in the meat evaporates, increasing the WHC [36]. In this study, therefore, it was determined that as the aging temperature drops, the evaporation of free water inside the dry-aged meat is inhibited, resulting in a gradual increase in WHC.

Table 5 shows the cooking yield and shear force of the beef loin dry aged using the electric field refrigeration system. The cooking yield showed an increasing trend as the aging period increased at all temperatures. Macharáčková et al. [37] reported that the cooking yield increased as the dry-aging period increased, which was similar to the result of this study. The correlation between WHC and cooking yield is determined by the amount of water present between the muscle fibers lost during the cooking process [38]. This reduces the loss of water through the chemical bond of the proteins of the fragmented cytoskeletons, resulting in a higher cooking yield [39]. Furthermore, since free water has already evaporated during the dry-aging process, the amount of water lost through cooking is small, and this study also confirms that the cooking yield increases along with the WHC.

The shear force showed the lowest value in the fourth week at 0 °C and −2 °C and in the second and fourth weeks at −1 °C (*p* < 0.05). Calpain, a proteolytic enzyme that affects shear force, is active at the beginning of aging and decreases as the aging period increases [40]. Calpains are active up to three weeks of aging on average, and afterward, because the calpains become less active, the shear force no longer increases or rises only slightly [41]. Furthermore, the water content affects the shear force, and in dry aging, the evaporation of free water in the meat occurs rapidly, while in the latter half of aging, the shear force does not decrease but increases due to the hardening of the meat [25]. Therefore, based on the WHC, cooking yield, and shear force results, we have determined that the tissues are softest when the meat is dry aged for four weeks when using the electric field refrigeration system.

### 3.4. TPC, TBARS and VBN

**Figure 1:**
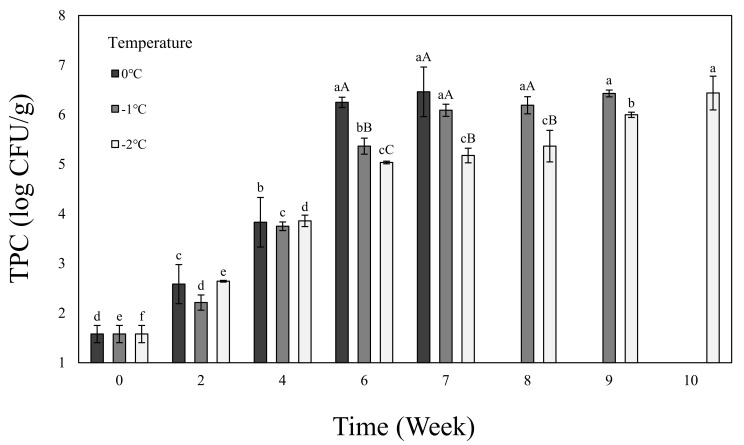
TPC of dry-aged beef loin on 0, −1, −2 °C temperature electric field refrigerate system. ^a–f^ Means in the same temperature with different letters are significantly different (*p* < 0.05). ^A–C^ Means in the same time with different letters are significantly different (*p* < 0.05). Samples were measured until aging stopped as the value of TPC reached 7 log CFU/g.

Figure 1 shows the TPC of the beef loin dry aged using the electric field refrigeration system. The TPC showed an increasing trend as the aging period increased at all temperatures versus a more gradually increasing trend as the temperature dropped. In the dry-aging process, micro-organisms are an important factor involved in the safety of food and the tenderness and flavor of meat, and they are affected by the aging temperature [42]. Microbial growth and proteolysis occur rapidly in meat at high temperatures, but slowly at low temperatures [9]. Furthermore, at low aging temperatures, the growth of Pseudomonas sp., psychrotrophic bacteria, affects TPC [43]. The microbial growth is a criterion for determining whether the meat is spoiled or not: If the TPC exceeds 7 log CFU/mg, it is determined that the meat is spoiled [44]. In this study, therefore, we determined that within a range not exceeding 7 log CFU/mg, the electric field refrigerator dry aging is most suitable for up to 7 weeks at 0 °C, 9 weeks at −1 °C, and 10 weeks at −2 °C.

**Figure 2:**
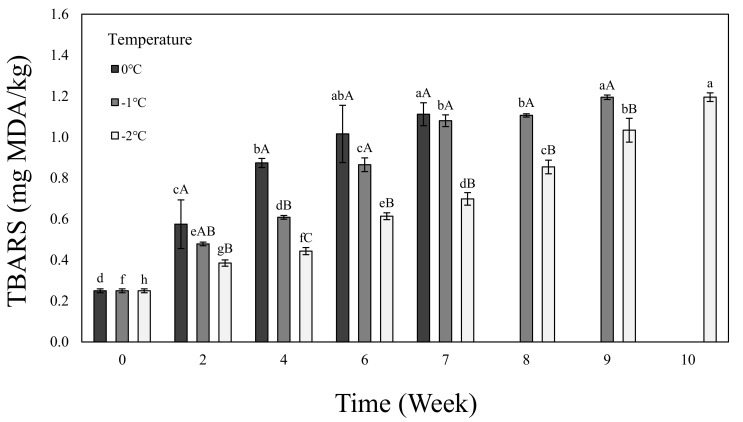
TBARS of dry-aged beef loin on 0, −1, −2 °C temperature electric field refrigerate system. ^a–^^h^ Means in the same temperature with different letters are significantly different (*p* < 0.05). ^A^^–^^C^ Means in the same time with different letters are significantly different (*p* < 0.05). Samples were measured until aging stopped as the value of TPC reached 7 log CFU/g.

Figure 2 shows the lipid oxidation degree (TBARS) of the beef loin dry aged using the electric field refrigeration system. The TBARS showed an increasing trend as the aging period increased at all temperatures and a more gradually increasing trend as the temperature dropped. Because dry aging is performed with exposure to the air, lipid oxidation occurs more rapidly than with other aging methods [45]. MDA, a reactive compound formed by lipid oxidation, spreads to sarcoplasmic proteins and interacts with myoglobin, causing discoloration [46]. The MDA production rate becomes slower as the aging temperature drops [47]. Considering that rancid flavor is a unique flavor of dry-aged beef, Ribeiro et al. [48] recommend 2.28 mg MDA/kg or less as a standard for TBARS. In this study, therefore, we determined that if meat is dry aged using the electric field refrigeration system, it will be safe in terms of quality and hygiene since the TBARS will not exceed 2.28 mg MDA/kg at all temperatures.

**Figure 3:**
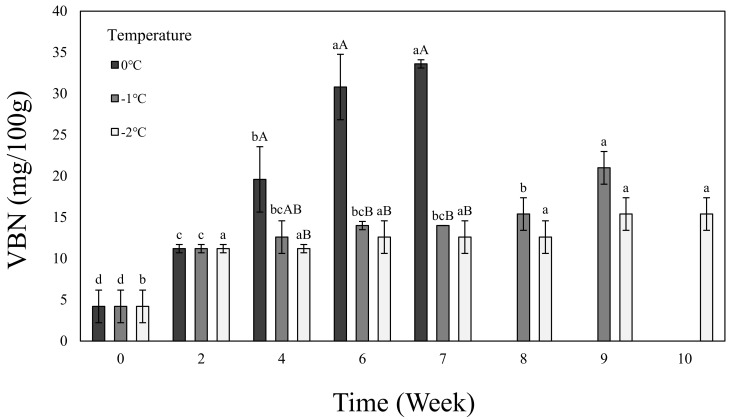
VBN of dry-aged beef loin on 0, −1, −2 °C temperature electric field refrigerate system. ^a–^^d^ Means in the same temperature with different letters are significantly different (*p* < 0.05). ^A, B^ Means in the same time with different letters are significantly different (*p* < 0.05). Samples were measured until aging stopped as the value of TPC reached 7 log CFU/g.

Figure 3 shows the VBN of the beef loin dry aged using the electric field refrigeration system. The VBN showed an increasing trend as the aging period increased and a more gradually increasing trend as the temperature dropped. The main cause of the VBN is amino acid deamination by proteolytic enzymes and the ammonia production by micro-organisms [49]. At low temperatures, the proteolysis and the microbial growth slow down, resulting in a gradual increase in VBN [50]. Furthermore, Lee et al. [51] have reported that storage at a temperature below the glass transition temperature may reduce the increase in the amount of volatile basic nitrogen. VBN has a positive correlation with TPC, and in the case of beef, it is known that the early stage of decaying occurs between 15 and 20 mg/100 g [52]. In this study, we determined that, within a range of VBN values that do not exceed 20 mg/100 g, meat dry aged using the electric field refrigeration system will be safe in terms of quality and hygiene for up to 4 weeks at 0 °C, 8 weeks at −1 °C, and 10 weeks at −2 °C.

## 4. Conclusions

This study investigated the physico-chemical (aging yield, trimming yield, pH, color, WHC, cooking yield, and shear force) and storage properties (TPC, TBARS, and VBN) of beef loin to establish the dry aging period in an electric field refrigerating system (temperature: 0, −1, −2 °C). Regarding its physico-chemical properties, with increasing dry aging period, aging yield, trimming yield, redness, yellowness, and chroma decreased, and pH, lightness, hue angle, WHC, and cooking yield increased. While shear force showed its lowest value at 4 weeks at all three temperatures. The results of storage properties showed that TPC and TBARS satisfied the standard values (TPC: 7 log CFU/mg, TBARS: 2.28 mg MDA/kg) during the dry aging period. However, VBN at 6 weeks at 0 °C and 9 weeks at −1 °C exceeded the standard value (20 mg/100 g). In conclusion, the dry aging periods when using an electric field refrigerating system must not exceed 4 weeks at 0 °C, 8 weeks at −1 °C, or 10 weeks at −2 °C. However, considering the texture, four weeks at all temperatures seems to be suitable for manufacturing soft dry-aged beef loin.

## Figures and Tables

**Table 1:** Aging yield and trimming yield of dry-aged beef loin on 0, −1, −2 °C temperature electric field refrigerate system.

Trait	Temperature (°C)	Time (Week)								SEM ^(1)^
		0	2	4	6	7	8	9	10	
Aging yield (%)	0	-	83.67 ^aA^	71.69 ^bA^	63.66 ^cA^	56.51 ^dA^				0.45
	−1	-	66.37 ^aB^	57.79 ^bC^	53.20 ^bcB^	48.45 ^cdB^	45.76 ^d^	45.08 ^dB^		0.48
	−2	-	79.48 ^aA^	65.93 ^bB^	54.72 ^cB^	49.74 ^dB^	49.24 ^d^	47.73 ^dA^	46.64 ^d^	0.25
Trimming yield (%)	0	-	74.04 ^aA^	58.90 ^b^	50.22 ^b^	30.49 ^cB^				1.08
	−1	-	56.46 ^aB^	54.52 ^a^	45.33 ^b^	35.53 ^cB^	31.91 ^cdB^	26.25 ^dB^		0.45
	−2	-	74.34 ^aA^	56.20 ^b^	49.13 ^c^	44.57 ^cdA^	43.20 ^cdA^	41.50 ^deA^	36.55 ^e^	0.35

^(1)^ Standard error of the means. ^a–e^ Means in the same row with different letters are significantly different (*p* < 0.05). ^A–C^ Means in the same column with different letters are significantly different (*p* < 0.05).

**Table 2:** pH of dry-aged beef loin on 0, −1, −2 °C temperature electric field refrigerate system.

Trait	Temperature (°C)	Time (Week)								SEM ^(1)^
		0	2	4	6	7	8	9	10	
pH	0	5.27 ^c^	5.58 ^bA^	5.60 ^abA^	5.75 ^abA^	5.86 ^a^				0.02
	−1	5.27 ^c^	5.60 ^bA^	5.62 ^bA^	5.67 ^bA^	5.68 ^b^	5.71 ^bA^	5.81 ^aA^		0.01
	−2	5.27 ^c^	5.44 ^bB^	5.47 ^aB^	5.49 ^aB^	5.50 ^a^	5.50 ^aB^	5.50 ^aB^	5.51 ^a^	0.01

^(1)^ Standard error of the means. ^a–c^ Means in the same row with different letters are significantly different (*p* < 0.05). ^A, B^ Means in the same column with different letters are significantly different (*p* < 0.05).

**Table 3:** Color of dry-aged beef loin on 0, −1, −2 °C temperature electric field refrigerate system.

Trait	Temperature (°C)	Time (Week)								SEM ^(1)^
		0	2	4	6	7	8	9	10	
CIE L^*^	0	39.38 ^c^	40.96 ^c^	41.60 ^bc^	44.02 ^abA^	46.43 ^aA^				0.31
	−1	39.38 ^d^	40.83 ^c^	41.13 ^c^	41.53 ^bcB^	41.83 ^bcB^	42.43 ^b^	46.23 ^aA^		0.09
	−2	39.38 ^d^	41.10 ^c^	41.70 ^bc^	42.07 ^bcAB^	42.23 ^bcAB^	42.43 ^bc^	42.90 ^bB^	45.85 ^a^	0.10
CIE a^*^	0	18.38 ^a^	14.68 ^bB^	10.24 ^c^	8.48 ^cdAB^	6.83 ^d^				0.29
	−1	18.38 ^a^	14.90 ^bB^	10.16 ^c^	6.27 ^dB^	5.33 ^de^	5.13 ^de^	4.37 ^e^		0.09
	−2	18.38 ^a^	17.10 ^aA^	13.30 ^b^	9.50 ^cA^	6.23 ^d^	5.55 ^d^	3.60 ^de^	2.80 ^e^	0.11
CIE b^*^	0	8.65 ^a^	7.33 ^b^	6.35 ^bc^	5.48 ^cd^	4.97 ^dA^				0.17
	−1	8.65 ^a^	7.43 ^b^	5.57 ^c^	2.97 ^d^	2.83 ^dB^	2.43 ^d^	2.23 ^d^		0.10
	−2	8.65 ^a^	7.55 ^ab^	5.95 ^b^	4.03 ^c^	3.87 ^cdAB^	3.73 ^cd^	3.67 ^cd^	2.07 ^d^	0.12
Hue angle	0	25.21 ^b^	27.50 ^b^	31.54 ^aA^	32.92 ^a^	35.55 ^aA^				0.42
	−1	25.21 ^bc^	26.50 ^ab^	30.41 ^aA^	25.39 ^bc^	23.68 ^bcB^	21.44 ^cB^	27.32 ^abB^		0.29
	−2	25.21 ^de^	24.44 ^e^	22.76 ^eB^	27.55 ^d^	31.74 ^cA^	33.17 ^cA^	50.18 ^aA^	45.82 ^b^	0.18
Chroma	0	20.31 ^a^	16.55 ^bB^	12.91 ^c^	10.18 ^dA^	8.14 ^dA^				0.29
	−1	20.31 ^a^	16.66 ^bB^	11.01 ^c^	6.94 ^dB^	6.32 ^dB^	5.71 ^de^	4.82 ^e^		0.08
	−2	20.31 ^a^	18.78 ^aA^	14.69 ^b^	10.39 ^cA^	7.34 ^dAB^	6.65 ^de^	4.54 ^ef^	3.00 ^f^	0.16

^(1)^ Standard error of the means. ^a–f^ Means in the same row with different letters are significantly different (*p* < 0.05). ^A, B^ Means in the same column with different letters are significantly different (*p* < 0.05).

**Table 4:** WHC of dry-aged beef loin on 0, −1, −2 °C temperature electric field refrigerate system.

Trait	Temperature (°C)	Time (Week)								SEM ^(1)^
		0	2	4	6	7	8	9	10	
WHC (%)	0	35.43 ^d^	61.91 ^cA^	80.82 ^bA^	90.22 ^abA^	95.82 ^aA^				0.88
	−1	35.43 ^f^	52.00 ^cAB^	58.36 ^dB^	68.69 ^cB^	89.69 ^bA^	91.50 ^b^	96.40 ^a^		0.19
	−2	35.43 ^g^	43.01 ^fB^	48.03 ^eC^	61.22 ^dB^	75.46 ^cB^	85.58 ^b^	97.51 ^a^	98.30 ^a^	0.17

^(1)^ Standard error of the means. ^a–^^g^ Means in the same row with different letters are significantly different (*p* < 0.05). ^A–C^ Means in the same column with different letters are significantly different (*p* < 0.05).

**Table 5:** Cooking yield and shear force of dry-aged beef loin on 0, −1, −2 °C temperature electric field refrigerate system.

Trait	Temperature (°C)	Time (Week)								SEM ^(1)^
		0	2	4	6	7	8	9	10	
Cooking yield (%)	0	70.76 ^c^	83.29 ^bA^	84.18 ^bA^	92.19 ^aA^	94.35 ^aA^				0.28
	−1	70.76 ^c^	77.88 ^bAB^	87.37 ^aA^	88.52 ^aB^	90.41 ^aB^	90.55 ^a^	91.52 ^aA^		0.27
	−2	70.76 ^e^	75.09 ^dB^	79.94 ^cB^	83.46 ^bcC^	86.04 ^abC^	86.90 ^ab^	87.76 ^abB^	90.10 ^a^	0.21
Shear force (N)	0	45.35 ^ab^	35.67 ^cA^	25.75 ^dA^	38.41 ^bcA^	47.30 ^aA^				0.66
	−1	45.35 ^a^	24.88 ^dB^	23.16 ^dB^	31.39 ^cB^	35.64 ^bcB^	37.70 ^b^	44.11 ^a^		0.42
	−2	45.35 ^a^	28.05 ^eB^	20.80 ^fB^	31.57 ^dB^	33.75 ^cdB^	35.21 ^c^	39.35 ^b^	42.55 ^ab^	0.20

^(1)^ Standard error of the means. ^a–f^ Means in the same row with different letters are significantly different (*p* < 0.05). ^A–C^ Means in the same column with different letters are significantly different (*p* < 0.05).

## Data Availability

Data sharing is not applicable to this article.
